# Evaluation of Superselective Transcatheter Arterial Embolization with n-Butyl Cyanoacrylate in Treating Lower Gastrointestinal Bleeding: A Retrospective Study on Seven Cases

**DOI:** 10.1155/2016/8384349

**Published:** 2016-07-27

**Authors:** Yuan Zhao, Gang Li, Xiang Yu, Ping Xie

**Affiliations:** Department of Radiology, Sichuan Medical Science Academy, Sichuan Provincial People's Hospital, Chengdu 610072, China

## Abstract

*Background*. To investigate the safety and efficacy of superselective transcatheter arterial embolization (TAE) with n-butyl cyanoacrylate (NBCA) in treating lower gastrointestinal bleeding caused by angiodysplasia.* Methods*. A retrospective study was performed to evaluate the clinical data of the patients with lower gastrointestinal bleeding caused by angiodysplasia. The patients were treated with superselective TAE with NBCA between September 2013 and March 2015. Angiography was performed after the embolization. The clinical signs including melena, anemia, and blood transfusion treatment were evaluated. The complications including abdominal pain and intestinal ischemia necrosis were recorded. The patients were followed up to evaluate the efficacy in the long run.* Results*. Seven cases (2 males, 5 females; age of 69.55 ± 2.25) were evaluated in the study. The embolization was successfully performed in all cases. About 0.2–0.8 mL (mean 0.48 ± 0.19 mL) NCBA was used. Immediate angiography after the embolization operation showed that the abnormal symptoms disappeared. The patients were followed up for a range of 2–19 months and six patients did not reoccur. No serious complications, such as femoral artery puncture point anomaly, vascular injury, and intestinal necrosis perforation were observed.* Conclusion*. For the patients with refractory and repeated lower gastrointestinal hemorrhage due to angiodysplasia, superselective TAE with NBCA seem to be a safe and effective alternative therapy when endoscopy examination and treatment do not work.

## 1. Introduction

Lower gastrointestinal (GI) bleeding is defined as bleeding distal to the ligament of Treitz, ranging from minor, self-limited bleeding to life-threatening hemorrhage [1]. GI bleeding may arise from multifactorial etiologies including diverticulosis, angiodysplasia (AD), neoplasm, and inflammatory bowel disease and it is most common in the elderly. Angiodysplasia is a small vascular malformation of the gut, prevailing in the right colon and cecum. It is a common cause of otherwise unexplained gastrointestinal bleeding and anemia and it increases in frequency with aging. Some cases present subtle blood loss, with the anemia symptoms predominating. Previous reports declared that 3–40% GI refers to angiodysplasia [2].

The less severe bleeding is fit for colonoscopy diagnosis which is adopted as the diagnostic modality of lower GI hemorrhage widely [3]. However, endoscopic therapeutic intervention only succeeds in a minority of patients [4–6]. The severe bleeding is better evaluated by angiography and managed with embolization. Angiography and embolization with particles are a microinvasive treatment option, which avoids the need for surgery and bowel resection. By using this method, the vessel supplying the angiodysplasia is selectively catheterized and embolized with microparticles. Angiography and transcatheter arterial embolization (TAE) of causative upper GI bleeding are widely accepted, but they have not achieved the same effect for lower GI bleeding [7, 8]. For example, TAE has the risk of intestinal ischemia necrosis for lower GI hemorrhage due to the lack of good artery anastomosis network of lower digestive tract [9]. As far as we know, there is very limited research on treating lower gastrointestinal bleeding caused by angiodysplasia using TAE.

The objective of the present study was to evaluate the clinical safety and efficacy of superselective TAE using n-butyl cyanoacrylate (NBCA) gum in treating lower GI hemorrhage caused by angiodysplasia. The patients with GI bleeding and AD were treated with superselective embolization using NCBA and were retrospectively studied. The clinical data including melena, anemia, and blood transfusion treatment were evaluated.

## 2. Patients and Method

### 2.1. Patients

This study was approved by the ethic committee of the hospital and the signed informed consent forms were obtained from all the patients. We performed a retrospective evaluation of clinical data that were collected on the patients who underwent TAE with NCBA between September 2013 and March 2015. The inclusion criteria were patients with chronic repeated gastrointestinal hemorrhage, failing conservative medicine treatment (intravenous administration of somatostatin for 3–5 days), contraindications to surgery, and positive digital subtraction angiography (DSA) with angiodysplasia. The exclusion criteria were multiple vascular malformation, obscure bleeding site, intestinal tumor, and contraindications to angiography.

The clinical symptoms were repeated: hematochezia, hemorrhagic anemia, low level hemoglobin (HGB), and multiple blood transfusions (more than three times). One case (Case 5) that was treated with argon plasma coagulation for intestinal angiodysplasia one year ago presented with gastrointestinal hemorrhage. One case (Case 6) had been treated with somatostatin and then presented with the above clinical symptoms. The other cases were treated with blood transfusion and other hemostasis before the transcatheter embolization.

### 2.2. Methods

The patients underwent gastrointestinal angiography and superselective embolization directed by DSA system (Philips Allura 15 and Siemens Artis zee). A 5 F vascular sheath was embedded through the right femoral artery puncture for abdominal artery angiography and mesenteric artery angiography. The contrast (24 mL) was injected in the speed of 8 mL/s under the pressure of 300 bpi. The characteristics of the bleeding artery were determined. Transcatheter embolization was performed with 1.7 F microtubule (Headway 17 Advanced Soft, Microvention TERUMO) which was put near the bleeding site. Injection of contrast agent by hand was performed to evaluate the injection speed and the volume of NCBA and prevent the backflow. NBCA was diluted with lipiodol by the ratio of 1 : 2 to 1 : 3 according to the distance between the catheter and the bleeding site. The concentration of NBCA was increased with the distance decreasing. After the microtubule was washed with 5% glucose liquid, NBCA embolization was performed in the sandwich approach. The injection speed and the used volume of NCBA were determined according to the previous injection of the contrast agent as described above. The injection was stopped if there was obvious backflow to the marginal artery. After the embolization, selective artery angiography was performed to evaluate the result.

The operator has clinical experience of 9 years in radioactive interventional therapy and is skilled in NBCA embolization for intracranial arteriovenous malformation, renal artery, hepatic artery, and hemorrhagic disease resulted from peripheral artery pseudoaneurysms.

### 2.3. Evaluation of Outcome and Follow-Up

TAE effect assessment refers to Society of Interventional Radiology (SIR) criteria [10]. Angiography was performed after the embolization. It was considered as a successful embolization that abnormal radiography signs, including abnormal vascular mass, early appearance of drainage veins, and contrast agent spillover, disappeared. The clinical signs including melena, anemia, and blood transfusion treatment were recorded. The valid treatment was defined as the following characteristics: no rebleeding, instable hemodynamics in 30 days, or visceral ischemia or necrosis. Rebleeding was defined as gastrointestinal hemorrhage with HGB decreasing >1 g/dL. The complications including abdominal pain and intestinal ischemia necrosis were recorded.

The patients were followed up to evaluate the efficacy in the long run. The melena, anemia symptoms, and blood transfusion were recorded.

## 3. Results

Seven cases (2 males, 5 females; age of 69.55 ± 2.25) met the inclusion criteria and were enrolled in the study. Two patients (Case 1 and Case 4) were combined with chronic renal failure. The mesenteric angiography and the celiac artery angiography showed vasodilation in the wall of digestive tract and early appearance of drainage veins. The hemorrhage lesions were located in jejunum (3 cases), the ileum (1 case), ascending colon (1 case), hepatic flexure of transverse colon (1 case), and descending colon (1 case).

**Figure 1:**
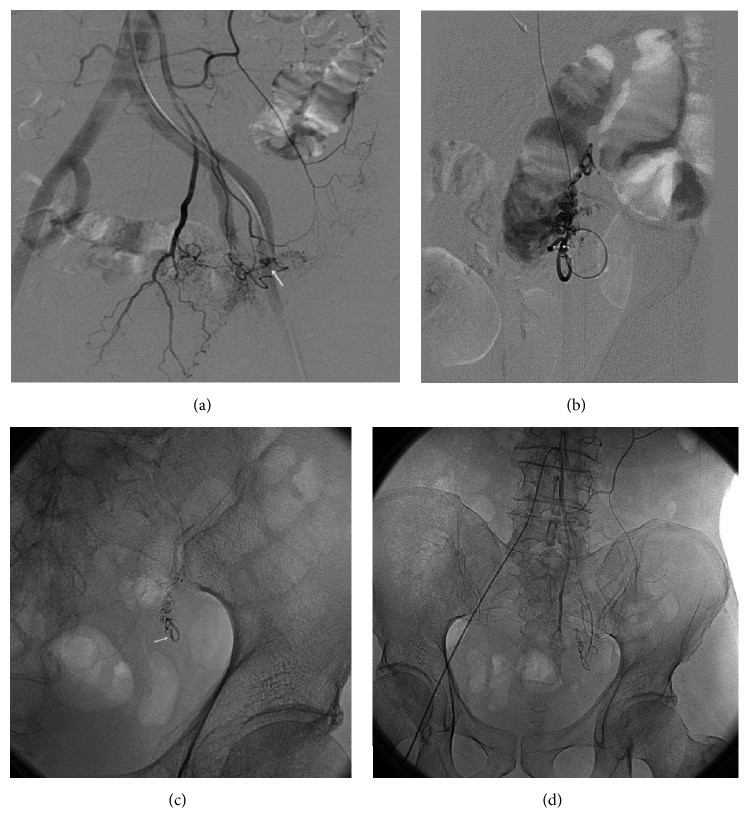
The radiography in the patient (Case 7) with lower gastrointestinal bleeding and angiodysplasia of sigmoid colon. (a) The preoperative radiography shows vascular malformation of sigmoid colon (as showed by the arrow). (b) The superselective radiography was used to evaluate the dose and velocity of the embolization agents. (c) It shows the deformed vascular mass (as showed by the arrow). (d) The postoperative radiography (without digital subtraction) showed that the abnormal sign disappeared.

**Figure 2 fig2:**
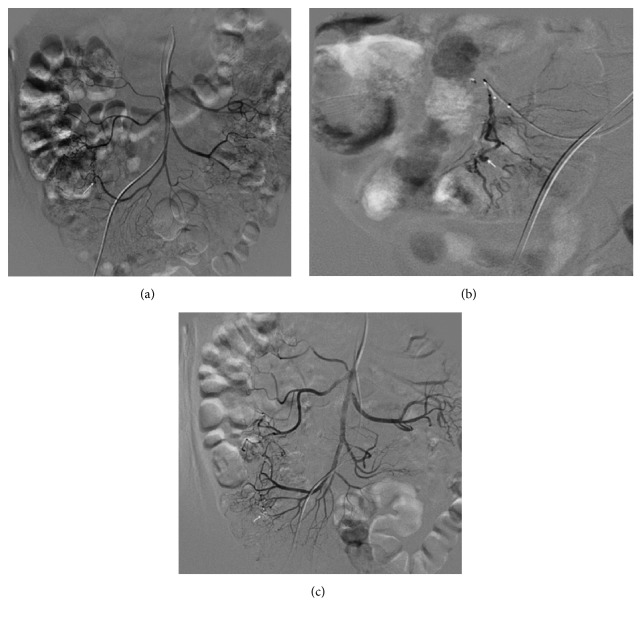
The radiography in the patient (Case 5) with lower gastrointestinal bleeding and angiodysplasia of ascending colon. (a) The preoperative radiography shows proximal vascular malformation of ascending colon (as showed by the arrow). (b) The superselective radiography showed the enlarged tortuous vessel and microaneurysm (as showed by the arrow). (c) The postoperative radiography showed that the abnormal sign disappeared (as showed by the arrow).

**Figure 3:**
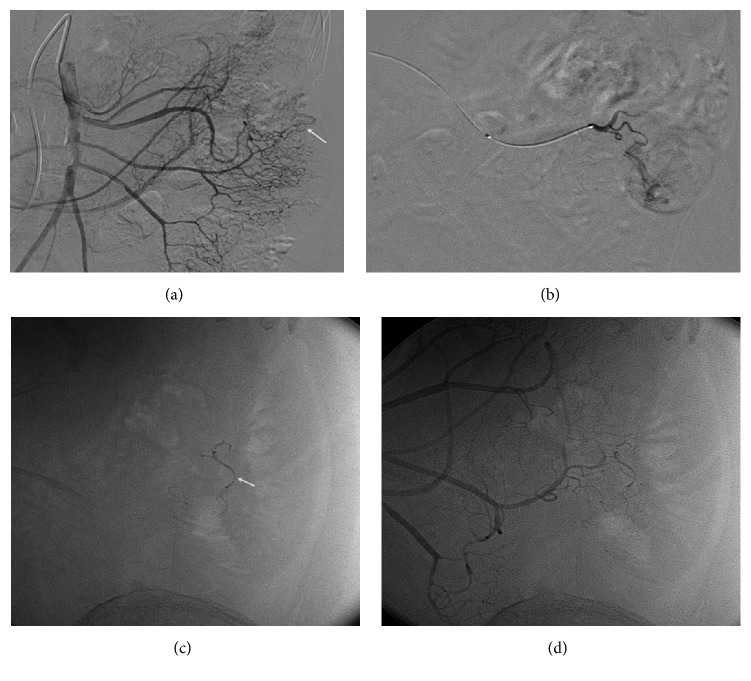
The radiography of the patient (Case 6) with lower gastrointestinal bleeding and angiodysplasia of jejunum. (a) The preoperative radiography shows vascular malformation of jejunum (as showed by the arrow). (b) The radiography in straight artery showed the enlarged tortuous vessel. (c) It shows vascular cavity cast after embolism (as showed by the arrow). (d) The postoperative radiography (without digital subtraction) showed that the abnormal sign disappeared.

The embolization was successfully performed in all cases (Figures 1 [Fig fig2]–3). One patient (Case 1) with vascular malformation of transverse colon underwent embolization procedures for two times considering that there were two straight arteries involved in supplying blood. NCBA used was 0.2–0.8 mL (mean 0.48 ± 0.19 mL). Immediate angiography after the embolization operation showed that the abnormal symptoms disappeared. An illustration of the therapeutic outcome was showed in Figure 1. All patients discharged from hospital three days postoperatively with normal stool color. Recurrent bleeding was not observed after the embolization operation and during the hospital stay.

The patients were followed up for a range of 2–19 months and six patients did not reoccur (Table 1). The gastrointestinal bleeding reoccurred in one patient (Case 4) with chronic renal functional failure 20 days postoperatively. The patient was treated with intestinal resection considering the early stage of the disease, intraoperative vasospasm, and incomplete embolism. As for the complications, one patient (Case 2) with jejunum lesions suffered temporary abdominal pains during embolization agent injection. One patient (Case 5) with terminal ileum diseases felt pain in the right lower abdomen but did not have rebound tenderness and muscular tension. The symptoms disappeared after conservative observation. No other complications, such as femoral artery puncture point anomalies, vascular injury, and intestinal necrosis perforation, were observed.

## 4. Discussion

In the present study, the seven patients with GI bleeding and AD were successfully treated with superselective embolization using NCBA. Gastrointestinal angiodysplasia is the pathologically expansive blood capillary between artery and vein. The mechanism may be the smooth muscle contraction of intestinal tract leading to the repeatedly low intensity of obstruction in the venule of intestinal wall. The increasing pressure in the capillary bed can induce functional failure of the anterior sphincter of capillary and forms the arteriovenous fistula finally [11]. Angiodysplasia accounts for 30–40% of the lower gastrointestinal hemorrhage of undetermined etiology [12]. Although most of gastrointestinal bleedings due to angiodysplasia are self-limitation, 10–30% patients have the possibility of rebleeding and the clinical performances are often chronic occult bleeding, recurrent episodes of melena, and iron-deficiency anemia which need high expensive [13].

Hemorrhagic angiodysplasia (HAD) treatment mainly aims to decrease anemia degree, transfusion times, and hemorrhage rate. Endoscope is the predominant therapy in treating HAD, but it limits the accurate location of petechial caused by abundant bleeding or bezoar. Moreover, because of different operation experience, the failure rate of endoscope diagnosis and treatment may reach 32% [14]. At present, iron supplements, octreotide, and thalidomide are the main clinical medicine. Swanson et al. reported that thalidomide alone or combined with oestrogen and progestin for treating HAD leads to relative poor consequence while endoscope and octreotide have not been proven adequately [15]. Enterotomy is another method to manage HAD. However, most of the patients with angiodysplasia are elderly and combined with various diseases; thus they have relative poor tolerance for surgery. The mortality rate of this surgery may reach 9%–47% [16]. Transartery infusion of pituitrin used to be an important method for interventional therapy of HAD; it almost is weeded out now because of the inevitably serious complications and higher bleeding rate made by pituitrin. Selective mesenteric angiography is needed when other minimally invasive or noninvasive ways cannot determine the bleeding site, because angiography can not only orientate the bleeding site, but also guide the embolization at the same time. Recent researches indicates that the successful rate of embolization to intestinal peripheral small arterial branch using microtubule is 80–100% with approximately 14%–29% bleeding recurrence rate [17]. Superselective embolization can avoid the extensive intestinal infarction and death, so most of researchers regard this method as a good choice for intravascular treatment.

The widely used embolic agents include microcoils, polyvinyl alcohol (PVA) particles, and gelfoam. The mechanism of embolization depends on the embolic agent used. Although the above embolization agents are effective to the majority, they still cannot get enough embolization effect for some patients, which may due to the fact that the embolization agent is unable to be transported to the bleeding site; bleeding in the collateral circulation; recanalization of the embolized vessel, especially for the patient with coagulation disorders [18].

PVA particles and gelfoam are unable to achieve the effect of precise embolism and may reversely flow to the nontarget vessel. NBCA can produce polymerization when exposed to blood or normal saline and then results in the permanent occlusion. This mechanical occlusion is independent of the thrombose ability of patients. The superselective embolization with liquid NBCA is able to embolize arteriovenous fistula, arterial vessel, and drainage vein at the same time. This complete embolization to the lesion site could reduce rebleeding that might be resulting from the establishment of collateral circulation, the incomplete embolism, or recanalization of the diseased blood vessel [6, 18]. Therefore, it is advantageous in treating the lower gastrointestinal hemorrhage. The liquid NBCA agent can be transported through slender microtube (0.01 inches), which can be used in the thin, twisty, and spastic vessels. What is more, the straight artery, terminal artery, and collateral vessels in the lesion area were embolized for the patients with GI bleeding and AD, which could avoid the GI bleeding reoccurrence.

Bowel necrosis is the most serious complication of embolization in treating the gastrointestinal hemorrhage. And it is correlated directly with the number of straight arteries that are embolized. Hence, it is critical to prevent this complication when performing superselective catheterization and embolization of the abnormal arterial vessels [19]. In the present study, 1.7 F microtube and 0.014-inch microwire were used and no such complications were observed. The 1.7 F nerve microcatheter can transmit NBCA but is unable to convey the 0.018-inch microcoil. PVA and gelfoam can easily block the 1.7 F microcatheter and they could not be clearly monitored by X-ray.

NBCA mixed with iodipin has commendable X-ray radiopacity and its concentration can be controlled expediently. The lower NBCA concentration follows with the better permeability to distal vessel. The usually used proportion of NBCA gum and iodipin is 1 : 2-1 : 3. The tip of microcatheter tip usually cannot reach the bleeding sites if the artery is slim, stenosis, or spasm, in which condition the NBCA gum can reach and embolize the bleeding site. Thus, NBCA decreases the rebleeding that is resulting from the collateral circulation. In the present study, the spasm occurred in 2 cases when the superselective catheterization reached the middle section of the straight artery; the 25% NBCA mixture (1mlNBCA: 3 mL UF) was used. The satisfactory embolization was achieved in the bleeding site that was distal to the tip of catheter. One patient, who had the medical history of percutaneous transluminal coronary angioplasty and the administration of antiplatelet drugs for a long term, was cured without reoccurring during the 18-month follow-up.

There are several limitations in the present study. Firstly, the sample size is very small and this is a retrospective study. Secondly, the follow-up period is short. In conclusion, for the patients with refractory and repeated lower gastrointestinal hemorrhage due to angiodysplasia, TAE with NBCA seems to be an effective alternative therapy when endoscopy examination and treatment do not work.

## Figures and Tables

**Table 1:** General information of the patients and the therapeutic outcome.

Case number	Age/gender	APTT (s)	PT (s)	INR	Previous medical history	NBCA : LUF	Lesion site in radiography (before embolization)	Follow-up time
1	56/M	27.2	11.7	1.01	Repeated hemafecia for 6 months, secondary anemia, admission to hospital 3 times	1 : 2	Hepatic flexure of transverse colon	2 months, nonbleeding
2	57/F	23.6	9.9	0.85	Repeated hemafecia for 7 months, secondary anemia	1 : 2	Jejunum	7 months, nonbleeding
3	70/F	23.3	11.3	1.01	Repeated hemafecia for 12 months, secondary anemia	1 : 2	Jejunum	12 months, nonbleeding
4	69/F	41.3	14.4	1.22	Repeated hemafecia for 6 months, secondary anemia	1 : 2	Descending colon	Rebleeding after 20 d
5	77/F	22.1	11.6	1.00	Repeated hemafecia for 12 months, secondary anemia, admission to hospital 3 times, APC	1 : 3	Ascending colon	17 months, nonbleeding
6	77/F	29.8	11.9	1.03	Repeated hemafecia for 12 months, secondary anemia	1 : 2	Jejunum	19 months, nonbleeding
7	90/M	25.0	10.1	0.87	Repeated stool occult blood for 2 months, progressive hemoglobin decrease after treatment	1 : 2	Colon sigmoid	18 months, nonbleeding

APTT: activated partial thromboplastin time, PT: prothrombin time, INR: international normalized ratio.
